# Rising temperature drives tipping points in mutualistic networks

**DOI:** 10.1098/rsos.221363

**Published:** 2023-02-01

**Authors:** Subhendu Bhandary, Smita Deb, Partha Sharathi Dutta

**Affiliations:** Department of Mathematics, Indian Institute of Technology Ropar, Rupnagar 140 001, Punjab, India

**Keywords:** climate warming, mutualistic communities, ecological networks, tipping points, community collapse

## Abstract

The effect of climate warming on species' physiological parameters, including growth rate, mortality rate and handling time, is well established from empirical data. However, with an alarming rise in global temperature more than ever, predicting the interactive influence of these changes on mutualistic communities remains uncertain. Using 139 real plant–pollinator networks sampled across the globe and a modelling approach, we study the impact of species’ individual thermal responses on mutualistic communities. We show that at low mutualistic strength plant–pollinator networks are at potential risk of rapid transitions at higher temperatures. Evidently, generalist species play a critical role in guiding tipping points in mutualistic networks. Further, we derive stability criteria for the networks in a range of temperatures using a two-dimensional reduced model. We identify network structures that can ascertain the delay of a community collapse. Until the end of this century, on account of increasing climate warming many real mutualistic networks are likely to be under the threat of sudden collapse, and we frame strategies to mitigate this. Together, our results indicate that knowing individual species' thermal responses and network structure can improve predictions for communities facing rapid transitions.

## 1. Introduction

The rate of increase in global temperature over the past 25 years is approximately four times greater than the rate of increase over the past 150 years as a whole [1]. An alarming rise in global temperature is one of the major aftermaths of human influence on climate that disrupts widespread population dynamics [2–5]. The consequences of such disruptions on species' abundances, interactions and community collapse are poorly understood [6–9]. Predicting species' responses to ongoing global warming is of great importance for the management and conservation of ecosystems. Until now, little is known about how increasing temperature influences species dynamics in complex communities [10–12]. A few recent studies highlight the effects of climate warming on food webs [13,14]. These studies confirm the complex changes along trophic levels caused by warming and eco-evolutionary feedbacks as a subsequent conservation policy to preserve biodiversity. Detecting the response of communities encompassing species of different genus connected in terms of cooperation and competition remains largely elusive [15–18]. Specifically, recognizing the effects of warming on the structure and function of mutualistic communities is crucial [19–23].

Mutualism is the ecological interactions between different species belonging to two distinct taxa cooperating for mutual welfare and services [24–27]. Mutualism has been vital for the survival of several species and an essential component of biogeochemical cycles, such as carbon and major nutrient cycles [28]. As mutualism binds a multitude of species to a common fate, benefiting them, it also holds these species at potential risk of extinction when exposed to a degrading environment [28–30]. Species in mutualistic networks, ranging from manufacturer–contractor to plant–pollinator networks, gain positive growth benefits, which in turn increases community productivity. Although mutualistic networks are widely heterogeneous in terms of interactions per species, they possess a well-defined connectivity distribution and structural pattern [24,31]. Plant–pollinator networks usually share a high degree of nestedness for a given connectance [32,33]. These structural properties have implications for the robustness of a mutualistic community [22,34,35]. Loss of resilience of mutualistic networks to extinction threats has been observed previously in the face of climate warming [20,22]. Recent studies have reported that nestedness, which ensures a cohesive core and asymmetric degree distribution, is mainly responsible for the stability of mutualistic networks [29,34,36,37]. In all probability, mutualistic networks have an optimal structure that maximizes ecosystem productivity and ensures network stability in deteriorating environmental conditions [23]. Nonetheless, recent studies by Miller-Rushing *et al.* [38] reveal changes in the phenological behaviours of forbs and bees with temperature variation. Also, others confirm the effect of habitat temperature on the phenological activities of various plants and pollinators, further resulting in changed physiological responses, loss of interaction and reduced population abundances [39]. Given the consequences of temperature variation on plants and pollinators, and the fact that previous studies have envisioned tipping points in mutualistic networks under acute disturbances [34,40], little is known about how a rise in temperature can alter species persistence. Hence, the current global warming, the variation in habitat temperature and a network’s structural properties that influence the proximity of a critical transition demand careful investigation.

Critical transition or tipping in an ecosystem is characterized by sudden, large, often irreversible and unexpected shifts from a steady state to another alternate steady state due to a parameter drifting [41–44]. While critical transitions are relatively easy to forecast when a leading species or a small number of species determine the state of an ecosystem, this is not the case for complex communities where interactions between many species determine ecosystem dynamics. Critical transitions can occur in mutualistic communities due to the positive feedback between mutually beneficial species; in plant–pollinator communities, a decline in pollinator abundances can negatively affect plant abundances, which in turn is bad for pollinators [6,29,34,45–47]. Mutualistic networks play integral roles in promoting species persistence and maintaining biodiversity in terrestrial ecosystems [28]; predicting tipping points in mutualistic networks due to increasing mean habitat temperature is critical.

Considering species’ biological rates and parameters as constants does not allow us to understand the effect of rising temperature on the stability of mutualistic networks, which—if ignored—could mean we miss out on information crucial to foresee a community collapse in such networks. To our knowledge, no theoretical/modelling studies have yet considered the effects of species' individual thermal responses on communities exhibiting mutualism. It remains unclear how species dynamics on being exposed to high-temperature conditions can trigger cascades of extinction, and thereby a community collapse in a mutualistic network [48,49]. Here, we investigate sudden transitions in 139 real-world mutualistic networks (from the Web of Life: Ecological Networks Database, http://www.web-of-life.es/) subject to varying degrees of temperature. We develop a mutualistic network model incorporating species' individual thermal responses. This is particularly important as mutualistic interactions between plant–pollinator communities involve plant-visiting ectothermic insects that are sensitive to temperature variations [50].

Here, we show the appearance of tipping points at high temperatures in plant–pollinator networks, where the network’s state abruptly shifts to an alternate state as the driver of pollination declines. Subsequently, to find the recovery path of the system post-collapse upon improving conditions, we focus on the correlation between climate warming and hysteresis, irrespective of the complexities (degree distributions, connectance, nestedness, etc.) in a higher dimensional system. Hysteresis is defined as the condition where multiple alternate stable states exist under similar environmental conditions and recovery to a previous alternate stable state is non-trivial (the point of recovery is not the same as the point of collapse). In other words, it implies that in the presence of positive feedback loops, the current state of the system depends on the historic state of the system. Further, we found that mutualistic plant–pollinator networks with an optimal structural property can withstand harsh warming conditions and exhibit increased resilience to perturbations. However, the required optimal structure for sustenance varies with the level of environmental deterioration. Also, a gradual change in the habitat temperature can alter species' phenologies and abundances, which may lead to different flowering timings, reducing pollination and resulting in loss of interactions or species. Therefore, we also consider the effects of species loss (removal of plants) or interaction loss (removal of pollinators, which is equivalent to removal of links between plants and pollinators) [51] attributed to various factors such as critically low abundance or functional dissimilarity between species exhibiting mutualism. We also determine the role of the generalist species in triggering a community collapse. Preventing the loss of such species has the potential to prevent or delay a community collapse at high temperatures. Stability analysis helps to understand the functioning of an ecological system, but this is theoretically challenging for a higher dimensional nonlinear system. We perform stability analyses of the reduced two-dimensional model using the dominant eigenvalue of the Jacobian matrix evaluated at the steady states, which allows understanding of the dynamics of the higher dimensional network with temperature variation [52]. Overall, our findings underline that efforts to mitigate climate warming and suitable conservation policies can manage the extinction risk of mutualistic communities.

## 2. Models and methods

We perform our analysis on real plant–pollinator networks differing in their structural properties (e.g. connectance and nestedness), dimensionality and species variety. These networks are also diverse depending on their geographical location and climatic zones. We employ the interaction matrices for these networks into a mutualistic network model. Existing mutualistic network models, often described by a set of first-order differential equations, represent the dynamics of plant–pollinator communities [29]. However, they do not include species' biological rates and parameters as temperature-dependent functions. Here, we develop a network model incorporating the influence of temperature on species' biological rates and parameters (i.e. growth rate, mortality rate and handling time). We denote *A*_*i*_ and *P*_*i*_ as the abundances of the *i*th plant and pollinator species of a network, respectively. The model representing a group of *S*_*A*_ pollinator species and *S*_*P*_ plant species has the following form:

2.1a $$\frac{dA_{i}}{dt}=A_{i}\left(\alpha_{i}^{\left(A\right)}(T)-k_{i}(T)-\sum_{\;j=1}^{S_{A}}\beta_{ij}^{\left(A\right)}A_{\;j}+\frac{\sum_{k=1}^{S_{P}}\gamma_{ik}^{A}P_{k}}{1+h(T)\sum_{k=1}^{S_{P}}\gamma_{ik}^{A}P_{k}}\right)+\mu_{A}$$

and

2.1b $$\frac{dP_{i}}{dt}=P_{i}\left(\alpha_{i}^{\left(P\right)}(T)-\sum_{\;j=1}^{S_{P}}\beta_{ij}^{\left(P\right)}P_{\;j}+\frac{\sum_{k=1}^{S_{A}}\gamma_{ik}^{P}A_{k}}{1+h(T)\sum_{k=1}^{S_{A}}\gamma_{ik}^{P}A_{k}}\right)+\mu_{P},$$

where $\alpha_{i}^{\left(P\right)}(T)$ and $\alpha_{i}^{\left(A\right)}(T)$ represent the temperature-dependent intrinsic growth rate of the *i*th pollinator and plant, respectively; *k*_*i*_(*T*) denotes the decay rate of pollinators; *h*(*T*) is the handling time; and *β*_*ij*_ represents the competition strength between species. *μ*_*P*_ and *μ*_*A*_ represent the immigration factor of plants and pollinators, respectively, and are incorporated in order to prevent underflow errors or allow re-establishment of otherwise extinct species. However, these terms do not influence the qualitative dynamics of the system [29,52–54]. Mutualistic interaction may be present or absent, and its strength is denoted by *γ*_*ik*_. $\gamma_{ik}^{P}$ and $\gamma_{ik}^{A}$ represent the strength of mutualistic interaction of the plant and pollinator, respectively. *γ*_*ik*_ is a function of the nodal degree *d*_*i*_ and takes the following form:

2.2 $$\gamma_{ik}=\epsilon_{ik}\frac{\gamma_{0}}{{\left(d_{i}\right)}^{\delta }},$$

where $\epsilon_{ik}$ represents the entries of the network interaction matrix (obtained from 139 real-world networks); $\epsilon_{ik}=1$ if a link is present between the *i*th plant and *k*th pollinator species, and is 0 otherwise. *γ*_0_ is the average mutualistic strength, and *δ* modulates the trade-off between the interaction strength and the number of interactions. No trade-off (i.e. *δ* = 0) is the case of mutualistic interaction strengths not influenced by the network structure. In contrast, a full trade-off (*δ* = 1) assumes that benefits attained by species from mutualism are independent of the network topology. In real scenarios, one often assumes a moderate *δ* value, and here, we consider *δ* = 0.5 for simplicity. $\beta_{ij}^{\left(P\right)}$ and $\beta_{ij}^{\left(A\right)}$ represent the competition strength between the *i*th and *j*th plant and pollinator, respectively, which behave synergistically with temperature beyond the optimum [55]. For simplicity, we consider the intraspecific competition *β*_*ii*_ = 1 and interspecific competition *β*_*ij*_ (*i* ≠ *j*) = 0 [53] throughout the study. However, in reality, *β*_*ij*_ can take any value in (0,1), i.e. intraspecific competition is higher than interspecific competition, and the sensitivity of our results to non-zero *β*_*ij*_ (*i* ≠ *j*) is presented in electronic supplementary material, appendix, section §S1, figure S1.3.

### 2.1. Dependence of species process rates and parameters on temperature

Based on empirical evidence, recent studies have confirmed species' biological rates (e.g.birth rate, death rate and parameters (e.g. handling time)) as functions of temperature varying in the range 0–40°C [10,56–59]. Here, we consider temperature-dependent species' growth rate *α*_*i*_(*T*) exhibiting a unimodal symmetric response represented by a Gaussian function [58,60]:

$$\alpha_{i}(T)=\alpha_{\mathrm{opt}}\;e^{-{\left(T-T_{0}\right)}^{2}/2\sigma_{\alpha }^{2}},$$

where *T*_0_ is the temperature at which the value of *α*_*i*_(*T*) is optimal and equals *α*_*opt*_. $\sigma_{\alpha }$ denotes the performance breadth. For simplicity, the intrinsic growth rates of the plant and pollinator species are considered equal [29,53,61]. The handling time *h*(*T*) of the pollinators, obeying Holling type-II functional response exhibiting a hump or a U-shaped relationship with temperature, can be represented by a Gaussian function [59]:

$$h(T)=h_{\mathrm{opt}}\;e^{{\left(T-T_{0}\right)}^{2}/2\sigma_{h}^{2}},$$

where *h*_opt_ represents the value of *h*(*T*) at the optimum temperature *T*_0_. *σ*_*h*_ denotes the performance breadth. The per capita mortality rate of pollinators *k*_*i*_(*T*) is observed to follow the Boltzman–Arrhenius relationship [58] and is formulated as follows:

$$k_{i}(T)=K_{\mathrm{opt}}\;e^{A_{K}(1/T_{0}-(1/T))},$$

where *K*_opt_ is the mortality rate at the optimum temperature *T*_0_ and *A*_*K*_ is the Arrhenius constant. A reduced pollinator population or an increase in pollinator mortality due to environmental factors is modulated by the term *k*_*i*_. The functional response curves for the species' birth rate, handling time, and death rate are plotted in electronic supplementary material, appendix, §S1, figure S1.1.

We study dynamics (collapse and the possibility of subsequent recovery) of the network model (2.1) when birth rate, death rate and handling time (see electronic supplementary material, appendix, §S1, figure S1.1) are exposed to rising temperature in the range 0–40°C, for a fixed average mutualistic strength *γ*_0_. We find the critical temperature *T* value for a specific *γ*_0_ at which the system undergoes an abrupt transition to an extinction state and thus estimate species tolerance at different *γ*_0_. By varying temperature gradually in the range 0–40°C in the forward (increasing) and backward (decreasing) directions, we examine the points of collapse and recovery, respectively. The difference in the points of collapse and recovery provides a metric to study the hysteresis among plant and pollinator populations. Next, we study the combined effect of the structure of a mutualistic network and rising temperature on influencing tipping points. We account for the effects of nestedness, connectance and mutualistic trade-off, on advancing or delaying tipping points for a gradual increase in the temperature. We calculate nestedness using the NODF (Nestedness metric based on overlap and decreasing fill) measure [29], modularity using the formula in [62] and connectance (for more details, see box 1: glossary).

Box 1. **Glossary.**

*Connectance (*C*)*: The ratio of realized interactions to all possible interactions, *C* = *L*/(*S*_*P*_ × *S*_*A*_), where *L* is the number of realized interactions (links connecting plants and pollinators), and *S*_*P*_ and *S*_*A*_ denote the number of plants and pollinators, respectively.

*Nestedness (N)*: A bipartite network (usually represented as a matrix) is said to be nested when components having a few items in them (locations with few species, or species with few interactions) have a subset of the items of components with more items. In other words, nestedness describes the extent to which interactions form ordered subsets of each other. We calculate nestedness using the NODF measure, defined as follows:

$$N_{\mathrm{NODF}}=\frac{\sum_{i<j}^{S_{P}}N_{ij}+\sum_{i<j}^{S_{A}}N_{ij}}{\left(S_{A}(S_{A}-1)/2)+(S_{P}(S_{P}-1)/2\right)},\;\text{with}\;N_{ij}=\frac{d_{ij}}{\min(d_{i},d_{\;j})},$$

where *N*_*ij*_ is the nestedness of the species pair *i* and *j*; *d*_*i*_ and *d*_*j*_ are the number of ones in rows *i* and *j*, respectively; and *d*_*ij*_ is the number of shared interactions between rows *i* and *j* (the so-called paired overlap). Ecologically, *d*_*ij*_ is the number of times that species *i* and *j* interact with the same mutualistic partner.

*Modularity (Q)*: The degree to which densely connected compartments within a network can be decoupled into separate clusters interacting more within themselves compared with interaction across clusters. For a bipartite network represented by the matrix *B*, the modularity metric is expressed as follows:

$$Q_{B}=\frac{1}{E}\left(\sum_{i\in A,j\in P}b_{ij}-\frac{d_{i}d_{\;j}}{E}\right)\delta \left(g_{i},g_{\;j}\right),$$

where *b*_*ij*_ is the element in *B* representing a link (i.e. *b*_*ij*_ = 1) or no link (i.e. *b*_*ij*_ = 0) between species *i* and *j*; *g*_*i*_ is the module that species *i* belongs to under a certain partition; *d*_*i*_ is the degree of species *i*; *δ* denotes Kronecker’s delta; and *E* is the number of interactions in the network.

*Nodal degree*: The degree of a node (here, plant and pollinator species). It is defined as the total number of relationships involving that node (species).

*Tipping*: A sudden, large and often irreversible transition of a dynamical system from one stable state to an alternate stable state in response to small stochastic perturbations.

*Tipping point*: A threshold value at which a dynamical system experiences tipping.

*First point collapse*: The point at which the abundance of at least one pollinator node undergoes extinction (i.e. the abundance of that species falls below 1 × 10^−2^).

*Final point collapse*: The point at which the abundance of all the pollinator nodes undergo extinction (i.e. the abundance of all the pollinators falls below 1 × 10^−2^).

Trade-off (*δ*-value) accounts for the effects of asymmetry. To understand the role of a network's structural properties on the plant–pollinator dynamics, we calculate the first point of collapse and final point of collapse (for details, see box 1: glossary) in the 139 plant–pollinator networks and plot them against their nestedness—a structural property in such networks known to have a stabilizing influence [29]. Hitherto, we have studied how climate warming affects the abundance of plants and pollinators; nonetheless, it also impacts the distribution of species as well as species interactions [63,64]. We investigate how mutualistic plant–pollinator networks with different nestedness respond to such perturbations incorporated through species loss and interaction loss. In our approach, we try to find if *γ*_0_ affects all species equally, or the loss of a core of generalists (species with higher connectivity) has predominant impacts over their specialist (species with lower connectivity) counterparts at higher temperatures. We remove a fraction *f*_*P*_ of plants and a fraction *f*_*A*_ of pollinators to account for the effect of such perturbations. All parameters including temperature range and variability in *γ*_0_ are chosen from previous studies [29,53]. The system is solved numerically using the fourth-order Runge–Kutta method with adaptive step size, and the model is run until a stationary state has been reached.

### 2.2. Dimension reduction of the network model

In a higher dimensional network model having multiple variables, finding the analytical expressions for equilibrium points and determining their stability might be cumbersome. The dimension-reduced model serves as a simplified framework allowing analytical tractability of the stable and unstable steady states of the plant–pollinator system. The calculated eigenvalues from the Jacobian matrix of the reduced model can give us a preliminary idea of the stability of the system for variation in mutualistic strength and increasing temperature. Further, this will guide us to the tipping point of the mutualistic plant–pollinator network in the *γ*_0_ − *T* space. Finding the unstable steady state (USS) and the regions of non-zero abundance of the USS is important to understand the collapse and recovery of the system post-collapse, upon improving conditions for different *γ*_0_ and *T* values. The point where the system jumps from stable steady state (SSS) to USS is the tipping point.

Hence, for the mathematical analysis of the mutualistic network model (2.1), we reduce it in two dimensions by considering the averaged value of mutualistic interaction strength among plants and pollinators [52,65]. The dimension-reduced model can be written as follows:

2.3a $$\frac{dA_{e}}{dt}=(\alpha (T)-k(T))A_{e}-\beta A_{e}^{2}+\frac{\langle \gamma_{A}\rangle P_{e}}{1+h(T)\langle \gamma_{A}\rangle P_{e}}A_{e}+\mu$$

and

2.3b $$\frac{dP_{e}}{dt}=\alpha (T)P_{e}-\beta P_{e}^{2}+\frac{\langle \gamma_{P}\rangle A_{e}}{1+h(T)\langle \gamma_{P}\rangle A_{e}}P_{e}+\mu ,$$

where *A*_*e*_ and *P*_*e*_ are the effective abundances of pollinators and plants, respectively; *α*(*T*) represents the temperature-dependent effective growth rate of the network; *h*(*T*) denotes the effective handling time; *β* represents the combined effect of intraspecific and interspecific competition; 〈*γ*_*A*_〉 and 〈*γ*_*P*_〉 denote the effective mutualistic strength of pollinators and plants, respectively; and *k*(*T*) is the species death rate in averaging sense; and *μ* represents migration effects for the species [52] (for further details, see electronic supplementary material, appendix, §S2). The dimension-reduction technique used here is limited by the inability to incorporate heterogeneity at the species level (in terms of parameters). However, it enables the study of nonlinear phenomena such as bifurcations [66], basin structures [67] and transient chaos [68,69], which is otherwise impossible in the higher dimensional network. As shown in electronic supplementary material, appendix, §S2, figure S2.1, the reduced system well depicts the qualitative dynamics of the model, including the dynamics near a tipping point.

## 3. Results

### 3.1. Temperature-driven sudden transitions in mutualistic networks

**Figure 1.**
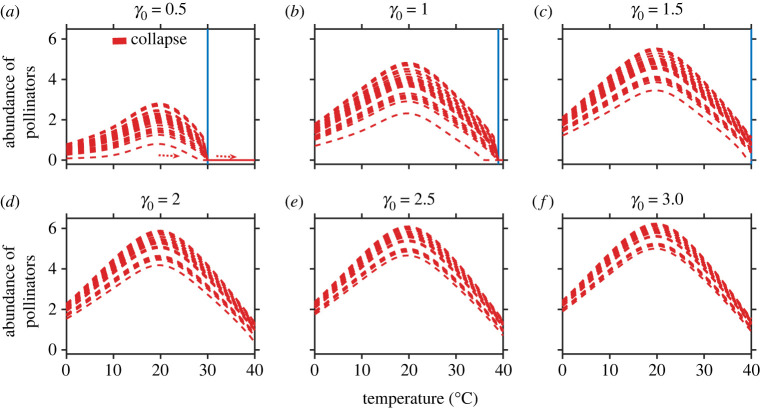
Higher temperatures can trigger catastrophic transitions at different interaction strengths (*γ*_0_): (*a*–*c*) On increasing the temperature in the range 0–40°C, for *γ*_0_ = 0.5 to *γ*_0_ = 1, the abundance of pollinators encounters catastrophic transitions. (*d*–*f*) At or beyond *γ*_0_ = 1.5, sudden community collapse is averted, although there is a gradual drop in population abundance with increasing temperature. The blue vertical line marks the occurrence of a critical transition. Each line in the sub-figures represents the abundance of each pollinator species in the network. The aforementioned result is obtained for network M_PL_006 with *S*_*A*_ = 61 and *S*_*P*_ = 17 (for details, http://www.web-of-life.es/). The taxonomic details of the aforementioned network are presented in electronic supplementary material, appendix, §S1, table S1.2. The parameter values are $\beta_{ii}^{A}=\beta_{ii}^{P}=1$, *δ* = 0.5, *μ*_*A*_ = *μ*_*P*_ = 10^−4^ and the other parameters $\alpha_{i}^{A}$, $\alpha_{i}^{P}$, *k*_*i*_ and *h* are obtained from their respective response function (unless stated, the network used and these values are the same in the rest of the figures).

We demonstrate the role of temperature in driving tipping points in mutualistic networks of varied dimensions, sampled across the globe. As depicted in figure 1, with a gradual change in temperature, a network can undergo a sudden transition from one stable state to an alternate stable state. However, this result changes with the strength of mutualistic interaction (*γ*_0_). The abundance of pollinators varies with changes in mean habitat temperature and can undergo an abrupt shift beyond optimum. With an increase in *γ*_0_, the threshold temperature at which the system collapses increases considerably. Further increase in *γ*_0_ beyond 1.5 prevents sudden community collapse in the considered feasible temperature range.

Our findings suggest that high temperatures can trigger critical transitions in a mutualistic community. This can otherwise be debilitated by lowering the degree of warming or preventing/delaying a transition by maintaining the requisite *γ*_0_. However, at a fixed *γ*_0_, striving to recover a species demands lowering the temperature by at least 2–3°C, which is extremely challenging in the face of rapid global warming. On the other hand, *γ*_0_ may be weak or strong depending on various global environmental factors [64,70], although direct relations are unknown. In line with the existing literature, we analyze the effects of both increases and decreases in interaction strength *γ*_0_ and their impact on network collapse at different temperatures.

**Figure 2.**
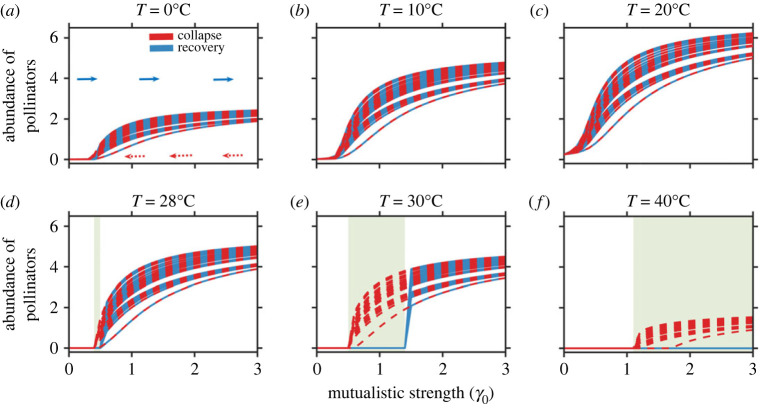
Catastrophic collapse in mutualistic networks for variation in mutualistic strength (*γ*_0_): (*a*,*b*) On decreasing the mutualistic strength *γ*_0_ in the range 3–0 (red), at low to moderate temperature until 20°C, the abundance of pollinators encounters a non-catastrophic transition. On increasing *γ*_0_ in the range 0–3, the system recovers to its previous state. (*c*) At 20°C, community collapse is averted, although there is a drop in abundance at low *γ*_0_ values. (*d*–*f*) As the temperature is further increased, a gradual shift transforms into a rapid collapse at a low *γ*_0_ value. As *γ*_0_ increases, striving to recover species (in the forward direction 0–3 (in blue)), a hysteresis loop formation is observed, and the width of this loop increases with an increase in warming. At very high temperature (40°C), the system does not recover. Results for other networks are presented in electronic supplementary material, appendix, §S1, figure S1.2.

As depicted in figure 2, for low mean habitat temperatures up to the optimum, the abundance of pollinators gradually collapses at comparatively low *γ*_0_ values. Beyond this, the community encounters sudden collapse, even at relatively high *γ*_0_ values. Likewise, increasing *γ*_0_ in the range of 0–3, it is observed that the system recovers early until optimum temperature, beyond which recovery is delayed. In the temperature range 32–40°C, the system fails to recover for any feasible *γ*_0_ value (0−3) and encounters one point collapse. We further observe that the points of community collapse and recovery are the same and change at and beyond 28°C. Clearly, the critical *γ*_0_ value that controls both the collapse and recovery of a system is influenced by the change in temperature (figure 2). To allow the system to recover, we increase *γ*_0_ in the direction 0–3, opposite to that which causes collapse. This leads to the formation of a hysteresis loop (figure 2*d*–*f*). The hysteresis loop is created first at approximately 28°C, being otherwise absent at lower temperatures. More explicitly, as shown in figure 2*f*, at 40°C, the system collapses at *γ*_0_ = 1.1 and does not recover on improving conditions post-collapse.

### 3.2. Temperature–network structure interplay influences tipping point

**Figure 3.**
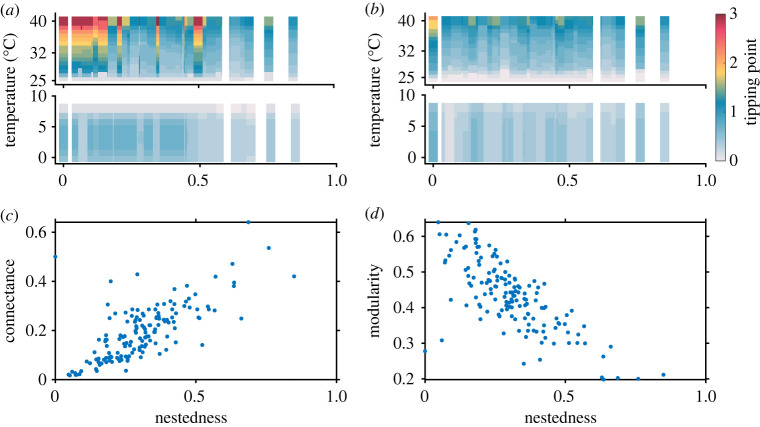
The role of network structure under varied degrees of warming in delaying the occurrence of a tipping point: (*a*) First point collapse of pollinators for 139 real plant–pollinator networks. At higher temperatures, nested networks undergo collapse at a considerably lower *γ*_0_ value (which marks the tipping point). (*b*) Final point collapse of the networks. The colours in the colour bar correspond to the *γ*_0_ values in the range 0–3, at which the system undergoes a first (*a*) and final (*b*) point collapse. Correlation between (*c*) connectance and nestedness, and (*d*) modularity and nestedness of 139 real networks is found. The results are averaged over 100 independent simulations.

In figure 3, we show results of 139 networks having nestedness varying in the range 0–0.84, allowing the networks to be exposed to a mean habitat temperature of 0–40°C. We find that nestedness plays a critical role in eluding the first point collapse (see box 1: glossary) at high temperature but has mixed effects on the final point collapse (see box 1: glossary). The tipping points for highly nested networks occur at substantially low *γ*_0_ values, while the collapse of networks with low nestedness occurs despite maintaining high *γ*_0_ values between species at extreme temperature. At low to moderate temperature, nestedness does not quite influence the tipping point. Figure 3 depicts the relation among *γ*_0_, nestedness and occurrence of tipping points at different temperatures. Tipping points and nestedness are majorly uncorrelated with temperature change, but exhibiting strong negative correlation beyond the optimum temperature (electronic supplementary material, appendix, §S1, figure S1.10). The methods for calculating nestedness of networks have discrepancies, and there remains a debate on the calculation of nestedness [32,62,71–73]. Nevertheless, nestedness could be correlated to other network properties such as connectance and network size, but for comparing our results with previous studies, we adopt the NODF measure [29]. Therefore, we analyze the effect of other structural properties of networks, e.g. connectance and network size, on the collapse of mutualistic networks with variation in temperature. The results presented in electronic supplementary material, appendix, §S1, figure S1.7(*a*) show that mutualistic networks with higher connectance experience delayed first point collapse and no prominent effect on the final point collapse (electronic supplementary material, appendix, §S1, figure S1.7(*b*)), consistent with our findings in figure 3 across different nestedness values.

Furthermore, we present first point and last point collapses of the 139 plant–pollinator networks, and show that no prominent effect is visible with variation in the number of plant and pollinator nodes (each plant or pollinator node represents a distinct plant or pollinator species) in a network (electronic supplementary material, appendix, §S1, figure S1.7(*c*)–(*d*)), unlike nestedness and connectance. Also as observed in electronic supplementary material, appendix, §S1, figure S1.8, connectance and nestedness in the considered 139 plant–pollinator networks—the two structural properties—are independent of the network size. Therefore, for the considered empirical data, either nestedness or connectance may be considered as a network's structural property for further theoretical explorations. In line with the existing literature, our results also suggest that nested networks are more robust to extinction than their random counterparts. Figure 3*c* shows a positive correlation between nestedness and connectance, while figure 3*d* shows that modularity is negatively correlated to nestedness. Thus, the results indicate high nestedness and connectance as structural properties of a mutualistic network, enabling them to withstand high temperatures and delay tipping points. Further, mixed effects of trade-offs are observed at low temperatures across networks (electronic supplementary material, appendix, §S1, figure S1.9).

### 3.3. Effects of species loss and interaction loss on network dynamics

**Figure 4.**
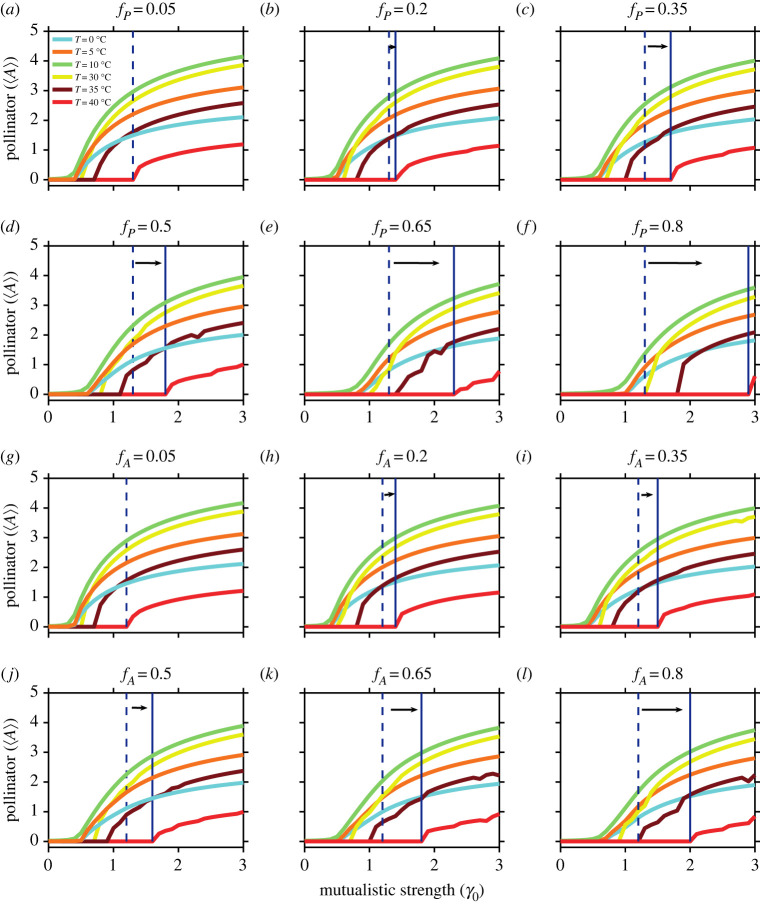
Effects of plant and pollinator loss for varied temperature regimes: the decline in average abundance of the pollinator community as a fraction of plants (*f*_*P*_) (*a*–*f*) and pollinators (*f*_*A*_) (*g*–*l*) are removed in decreasing order of their degree at temperatures ranging over the interval 0–40°C (denoted by different colours in the legend). As *γ*_0_ decreases from 3 to 0, the point of collapse advances as *f*_*P*_ and *f*_*A*_ are increased further. Plant loss has a more prominent effect on the collapse of the pollinator community, as indicated by the distance between the dashed and solid vertical lines for respective *f*_*P*_ and *f*_*A*_ values. A large difference is observed in tipping points at high temperature (40°C) for increases in *f*_*P*_ = 0.8 and *f*_*A*_ = 0.8. The dashed lines in (*a*–*l*) represent the point of collapse for *f*_*P*_ = *f*_*A*_ = 0.05 at 40°C. The solid lines in (*b*–*f*) and (*h*–*l*) represent the point of collapse for different values of *f*_*P*_ and *f*_*A*_ at 40°C, as mentioned above each sub-figure. 〈*A*〉 denotes the average abundance of pollinator species.

In figure 4, we study the impact of the more functional plant and pollinator loss on a network. In figure 4*a*–*f*, we observe that on the removal of a fraction of a species (plant loss) [51] in decreasing order of degree, there is negligible variation in the point of collapse at low to intermediate temperatures. On the contrary, at higher temperature (28°C and above) as a larger fraction of generalists (both plants and pollinators) are deleted, the point of collapse of the largest fraction precedes that of the smallest by a considerable amount. In figure 4*g*–*l*, we study the effect of small link perturbations (fraction of pollinators removed) on the network at various habitat temperatures (0°C, 5°C, 10°C, 30°C, 35°C and 40°C). We observe that the system is resilient to infinitesimal perturbations. However, on further increases in interaction loss, community collapse is elevated at 40°C. Our results indicate that effects of both interaction loss and species loss are more profound at extreme warming conditions.

### 3.4. Stability analysis of the network model via dimension reduction

In this section, after reducing the temperature-dependent mutualistic network model into a two-dimensional system, we study its dynamics. Mutualistic networks are composed of a large number of species leading to a high phase-space dimension. Analysis of this higher dimensional model requires insight into the underlying dynamics. This requires incorporating the necessary interactions by applying the weighted approach of obtaining the ensemble average value of *γ*_0_ (electronic supplementary material, appendix, §S2).

**Figure 5.**
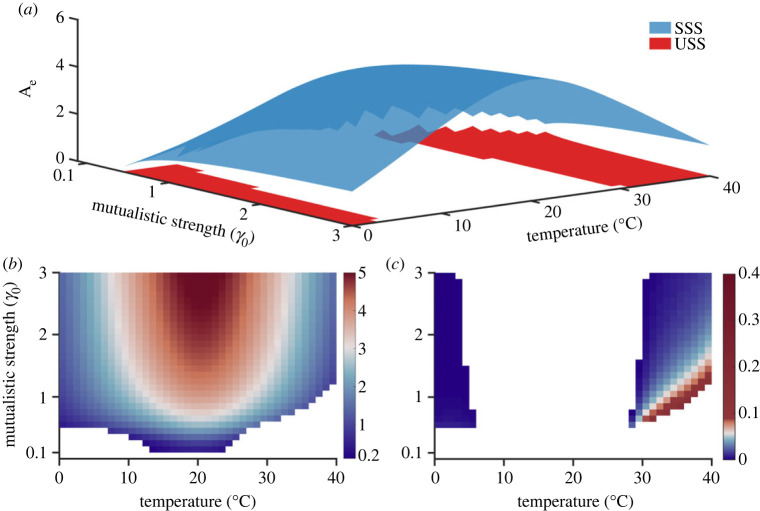
Stable and unstable steady states of the reduced model. (*a*) The stable (blue) and unstable (peach) steady-state surfaces obtained from the reduced model. The ensemble pollinator abundance is plotted as a function of *γ*_0_ in the temperature range 0–40°C. The 2D projections of the above stable and unstable surfaces depict the stable (*b*) and unstable (*c*) regions at different temperatures. In both the sub-figures, the colour in the colourbar denotes the abundance of the steady states (stable and unstable). SSS and USS denote stable and unstable steady states, respectively.

Figure 5 presents the average abundance of the pollinator species of the SSS and USS. We observe that the non-trivial SSS extends over the entire temperature for *γ*_0_ varying in the range 0.5–3. However, the system is unstable for very high (28–40°C) and low (0–8°C) temperatures. The two-dimensional projection reveals that the abundance corresponding to the stable branch is obtained for the maximum range of considered *γ*_0_ in a closed neighbourhood of the optimum temperature (figure 5). At higher and lower temperatures, the *γ*_0_ region corresponding to the SSS is reduced alongside reduced steady-state abundance compared with the intermediate temperatures (figure 5*b*), and the area is decreased further as we move away from the optimum temperature. In figure 5*c*, we observe non-zero pollinator abundance in the USS for higher temperatures and beyond a threshold *γ*_0_ (which increases with the increase in temperature), indicating an increased chance of tipping at high temperature across a range of *γ*_0_ values, unlike zero abundance for temperatures lower than the optimum. It is evident that species' biological responses to temperature are a key factor that governs the stability of mutualistic networks. While increased *γ*_0_ appears to hold back the system in the stable regime until a threshold temperature is reached, the system loses its stability whenever *h*(*T*) > *α*(*T*) and *k*(*T*) > *h*(*T*). Thus, the persistence of species is more favourable when the species' growth rate is much higher than the resource limitation rate and handling time is more than the decay rate, indirectly being controlled by the degree of warming.

**Figure 6.**
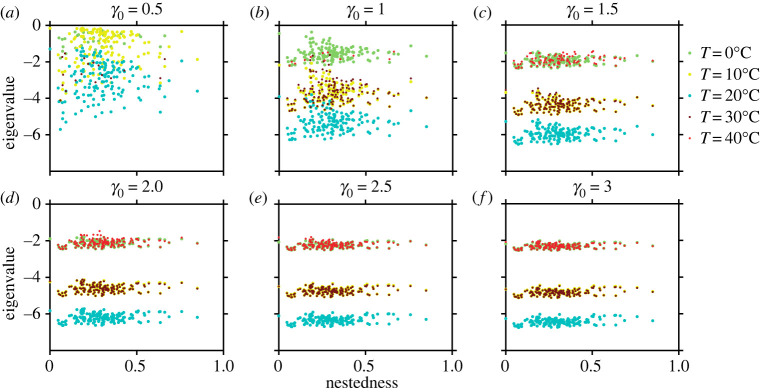
Effect of temperature and network structure on the eigenvalue of the SSS: (*a*–*f*) the eigenvalue of the Jacobian matrix corresponding to the non-trivial steady state plotted against the nestedness value for all 139 plant–pollinator networks for a fixed *γ*_0_ mentioned earlier in each panel. Each dot represents a network, with the colour denoting the respective temperature. The system is always more stable at optimum temperature; stability at higher temperature increases by increasing *γ*_0_, although the critical eigenvalue saturates at *γ*_0_ = 2 and does not increase further.

Distinctly around the optimum temperature, the system is more stable, as indicated in figure 6. Eigenvalues are more negative corresponding to the Jacobian of the SSS around the optimum temperature for all *γ*_0_ (electronic supplementary material, appendix, §S2, figure S2.2). The behaviour remains consistent across all 139 networks with varying structural properties (figure 6). While an increase in *γ*_0_ leads to a more negative dominant eigenvalue, the effects cannot be isolated to the optimum temperature alone and are minimal beyond *γ*_0_ = 2. However, at lower *γ*_0_ (*γ*_0_ = 0.5), no trends are observed. Our results suggest that high mutualistic strength is a means to maintain network stability at higher temperatures.

### 3.5. Management policies for species sustainability at higher temperatures

Envisioning the increased rate of global warming and predicting the fate of the real mutualistic networks, it is now vital to formulate practical management policies to avoid harmful consequences. The development of realistic mitigation policies requires model parameters varied across a range that agrees with the biological constraints. When considering such constraints, only a few management strategies are realizable in effect. Some of the cost-effective, viable principles involve maintaining the abundance of an influential pollinator or abating the decay rate of the same to a bare minimum [53]. Here, we demonstrate how inherent network structures interplay with the degree of warming to avoid tippings.

**Figure 7.**
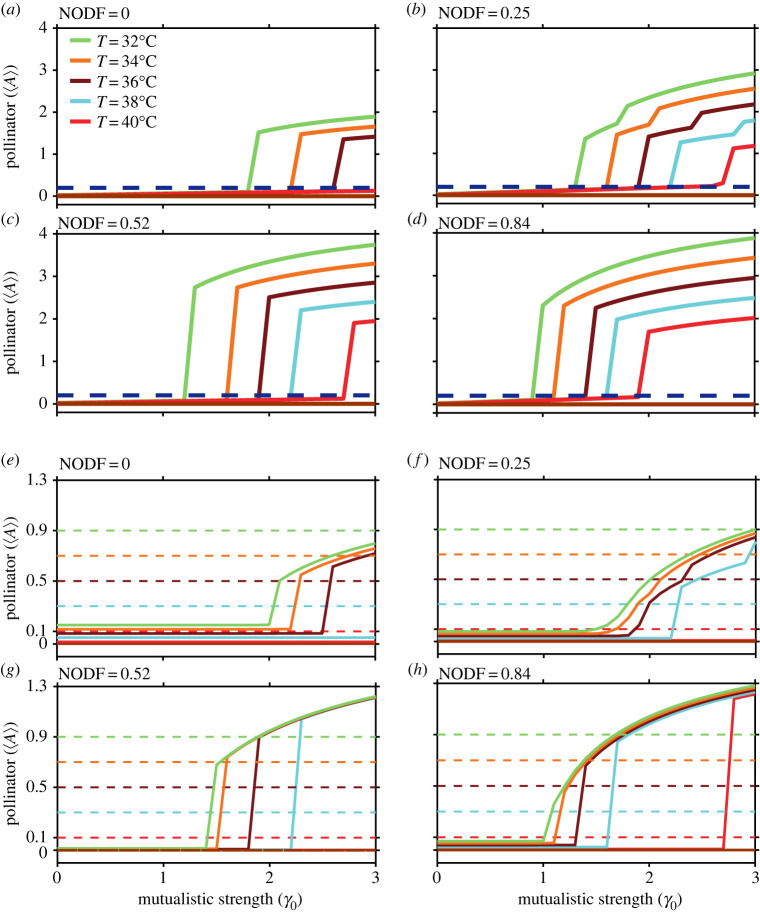
The role of network structure in managing tippings in mutualistic networks: (*a*–*d*) abundance management in four different networks in increasing order of nestedness (NODF). The dashed line (blue) denotes the fixed abundance of the generalist species as a management strategy, and the solid line (maroon) denotes the average abundance of species in the absence of management strategy at and beyond 32°C. The average abundance of the community is plotted at various temperatures above 32°C. Systems with higher nestedness experience early recovery for all temperatures in the range 32–40°C. At 40°C, network A (NODF = 0) fails to recover with the generalist species' abundance fixed at 0.2, while networks B (NODF = 0.25), C (NODF = 0.52) and D (NODF = 0.84) recover. Network D undergoes the fastest recovery. (*e*–*h*) The dashed lines denote the abundance of the generalist species fixed at abundances of 0.1, 0.3, 0.5, 0.7 and 0.9, and the recovery of the community at 40°C is correspondingly plotted in the same colour with a dashed line. The solid line (maroon) represents the average abundance of realized networks without any control. At 40°C, network A (NODF=0) fails to recover with the generalist species' abundance fixed at 0.3 or less, while networks B (NODF=0.25) and C (NODF = 0.52) do not recover with the generalist species' abundance fixed at 0.1 or less. But network D (NODF = 0.84) recovers with the generalist species' abundance fixed at any value greater than or equal to 0.1. We observe fixed threshold abundances of the generalist pollinator at 40°C for networks A, B, C and D, which aid in the system’s recovery.

We study how maintaining the abundance of the most generalist pollinators to a constant value can aid in the early recovery of the mutualistic community, which would have otherwise remained extinct. We have shown our result taking four different networks: network A (M_PL_061_33, *S*_*A*_ = 6, *S*_*P*_ = 2, NODF = 0), network B (M_PL_061_14, *S*_*A*_ = 11, *S*_*P*_ = 6, *NODF* = 0.25), network C (M_PL_006, *S*_*A*_ = 61, *S*_*P*_ = 17, NODF = 0.52) and network D (M_PL_059, *S*_*A*_ = 13, *S*_*P*_ = 13, NODF = 0.84). Simulations of the four different networks reveal that nestedness has a critical role in steering the recovery of species at higher temperatures. We observe that without any mitigation, the networks do not recover beyond 32°C irrespective of their underlying structure. On fixing the abundance of the influential pollinator at 0.2, these systems with distinct underlying structures recover for the aforementioned networks. The point of recovery is a latent function of the complex interaction of the habitat temperature and structural properties. As the mean habitat temperature is increased beyond 32°C, we find that networks with high nestedness recover at a decreased *γ*_0_ value. At 40°C, network A with nestedness (NODF) value 0 does not recover despite preserving an abundance of the generalist species, while networks B and C can be retrieved on further increasing *γ*_0_ values, and network D with nestedness value 0.84 recovers at *γ*_0_ = 1.9 (figure 7). In figure 7*e*–*h*, we observe that at 40°C, when there is a surge in the species decay rate, the threshold abundance of the generalist species needs to be increased. An increase in the fixed abundance of the generalist species will aid in the recovery of the community at a reduced *γ*_0_ value. This recovery point is further enhanced for a highly nested network, yet relationships are highly nonlinear. An important observation includes the fact that an average *γ*_0_ for low to moderately nested networks is essentially greater than, or comparable to, intraspecific competition (*β*_*ii*_ = 1) promotes recovery.

**Figure 8.**
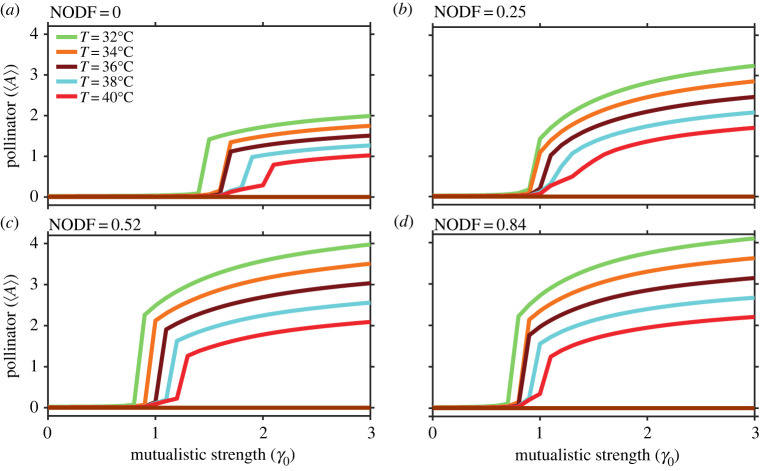
Managing tipping by minimizing the decay rate of the generalist species: at various temperatures above 32°C, the death rate of the generalist species for networks A, B, C and D is set at 0.01. The solid horizontal lines (maroon) represent species' abundance in the absence of a management strategy. For network A (NODF = 0), recovery is considerably delayed at the extreme temperature (40°C).

Another acceptable strategy is where the highest-degree (i.e. generalist) pollinator is targeted, and factors leading to its decay are minimized by setting its decay rate (*k*_*i*_) to 0.01, while other pollinators have a high decay rate. Figure 8 represents the average community trajectories at different temperatures above 32°C, at which the networks experience global extinction. Nestedness plays an equivalent vital role in expediting recovery at high temperatures, with a profound effect at the extreme. Despite the intraspecific competition, as the level of nestedness increases, pollinators interact more with generalist plants. Pollinators forming a part of a nested network tend to hold back the community on the verge of collapse and promote early recovery. Therefore, viable control principles should also involve adaptive strategies to increase nestedness of mutualistic communities alongside reducing environmental stress.

## 4. Discussion

Studies concerning the effect of species’ individual thermal responses of life history traits on ecological networks, such as mutualistic networks, remain less studied. Collected data of plants and their pollinating insects from Illinois, USA, in the late 18th century and revisited in 2010 reveal a loss of pollinator functioning and reduced adherence to their mutualistic counterparts [48]. Such information impels the study of network dynamics as species’ individual growth rate, birth rate and handling time are known to change with the degree of warming. Owing to the fact that a recovering species/community post-collapse demands lowering the temperature by up to a few degrees or increasing mutualistic strength among mutualists, our explorations revolve around the variations in the latter with network structural properties.

While an increase in temperature can push mutualistic communities to the brink of collapse (figure 1), increasing *γ*_0_ aids in species rescue and prevents community collapse (figure 1*d*–*f*). We find an interesting yet alarming fact that as *γ*_0_ is reduced, an increase in temperature beyond 28°C sets off catastrophic transitions (figure 2). A hysteresis loop is built up, which explains the increased net effect of mutualistic species on one another at high temperatures. These results are generalized and hold true for networks of varied size and structural properties (electronic supplementary material, appendix, §S1, figures S1.4 and S1.5). In the context of the robustness of our results—although, for simplicity, we assume an equal coefficient and symmetric functional response curves—qualitative results remain similar when considering functional response curves distributed randomly around a mean with a given variance (electronic supplementary material, appendix, §S1, figure S1.2) or skewed species' response rates. While perturbations in species' functional rates produce fluctuations in the abundance of pollinators without affecting the tipping point, skewed response curves shift the tipping temperature for a considered *γ*_0_; likewise, incorporating non-zero interspecific competition (*β*_*ij*_ ≠ 0), the requisite *γ*_0_ that aids in preventing species extinction increases (electronic supplementary material, appendix, §S1, figure S1.3). If one assumes *α*_*i*_(*T*)/*β*_*ii*_=*K*_*i*_(*T*), where *K*_*i*_(*T*) is the temperature-dependent carrying capacity, then it opens the possibility of further explorations considering a variety of shapes for *α*_*i*_(*T*) following the study of [59]. Interestingly, mutual dependence is a two-sided coin. At high habitat temperatures, as *γ*_0_ decreases, a sudden decline in plant abundance can trigger the extinction of pollinators and vice versa. However, an increase in mutualistic strength between the interacting species can aid in the recovery of both. Although we envision tipping points, gaining insight into the dynamics of the higher dimensional network model is rather difficult. Under this backdrop, the study of three minimal network models provides useful inferences: adding more plants into a network with a fixed number of pollinators can be a management policy to prevent pollinator collapse in the face of climate warming (electronic supplementary material, appendix, §S1, figure S1.6).

In a deteriorating environment, as the driver of pollinator decline increases and species or interaction loss becomes inevitable, preserving the key species can prevent community collapse. Removal of a fraction of generalists (figure 4) has a more harmful impact on the network compared with their specialist counterparts or random removals of plants or pollinators (electronic supplementary material, appendix, §S1, figure S1.11–S1.13) at high temperatures, since it is connected with a greater number of species. Designing conservation strategies to prevent network collapse demands lowering the degree of climate warming or maintaining the requisite network structural properties. Network collapse may be averted or delayed by preserving generalist species.

Our results have implications for fostering network resilience at high temperatures for the 139 real plant–pollinator networks. An intriguing result is that high nestedness (and low modularity) delays collapse or allows the system to recover early after being perturbed at extreme temperatures (37–40°C) (figure 3). This allows us to claim that maintaining the optimal structure can avoid issues related to network collapse due to anthropogenic stress. Managing networks at a high temperature by maintaining constant abundance or minimizing the decay rate of the most generalist pollinator is more straightforward for networks with higher or moderate nestedness values as they recover even with a low mutualistic strength. With regard to the feasibility of such techniques for empirical networks, artificial experiments need to be performed in the laboratory. One such experiment has been able to increase the connectance of plant–pollinator networks [74]. A field experiment was conducted to artificially increase the connectance of a mutualistic network by controlling the attractive power of plants in a bee–plant interaction network. The floral charisma and health of the experimental plants were maintained by means of chemical fertilizer. Improved pollinator statistics were observed in terms of richness, abundance and connectance in the fertilized plots compared with the unfertilized ones, indicating that requisite network properties can be attained or maintained through controlled experiments. In the absence of any management policy, networks—irrespective of their structural properties—do not recover at 40°C (figures 7 and 8). We also demonstrate this by the stability analysis of the reduced temperature-dependent two-dimensional model (electronic supplementary material, appendix, §S2, figure S2.2). As observed, the region of stability reduces, i.e. the system remains unstable for a large range of *γ*_0_. Hence, small environmental perturbations can push the system to an alternate extinction state. It may be noted that the interrelations between nestedness and modularity can differ across different mutualistic networks, and modularity does not always have a negative effect unlike our set of empirical networks [62].

Our study is limited by the fact that the mutualistic strength *γ*_0_ is not a function of temperature. Primarily, the mutualistic strength *γ*_0_ is not considered a function of temperature due to lack of empirical evidence related to its functional form. However, warming can cause changes in species' phenology—for instance, flowering times may differ as warming can lead to early flowering and pollinators' phenology will also change accordingly—resulting in mismatches, which decrease interaction strengths [75,76]. Nevertheless, *γ*_0_ indirectly accounts for the effect of changing temperature via the abundances of plants and pollinators. Therefore, our study provides a framework on which to build a more realistic model and has the potential to answer questions related to conditions that support partnership and improved predictions in the face of global warming. While our study revolves around the effects of gradual temperature change on mutualistic plant–pollinator communities, there remains a few open questions. An interesting possible future direction includes studying the effects of extreme climate events such as floods, droughts and erratic heatwave days over a period of time on an assembly of mutualistic communities. This may be investigated by incorporating infrequent sudden, large stochastic perturbations in the mean habitat temperature of the system [77,78]. Another promising direction is to study the temperature-driven network dynamics and the evolution of species while they exhibit niche-based interactions with their mutualist counterparts [79]. The modified framework would also allow understanding of the effects due to various factors such as anthropogenic changes and invasive alien species. As studied by [31], acknowledging adaptive behaviour can alter stability relations. Modelling the adaptive foraging of pollinators on plants with increasing climate warming, via a differential equation within the present framework, will be of substantial ecological importance. One may consider different temperature-response curves for juvenile and adult species, and study under the framework of stage-structured mutualism [80]. This is important yet challenging and requires visiting sites, collecting data and finding the functional curves, which might demand continuous monitoring of sites at regular intervals. Our study shows that behaviours at the individual level beget collective system-level consequences. Once network sites are revisited and different species' functionality with temperature variations are closely monitored, our results can be tested further to provide valuable insights into other real ecological networks.

As a starting point, while we only focus on the linear stability analysis of the reduced model for analytical tractability, computing the quasipotential of the higher dimensional plant–pollinator system is one practical tool for quantifying the stability of the higher dimensional system. Determining the stability of a stochastic mutualistic plant–pollinator network using a quasipotential function [81,82], though challenging, is an important future direction and will provide more insight into the dynamical behaviour of the system. While our study confirms that increasing temperature can affect tipping points in mutualistic networks, species’ phenological traits also evolve in response to warming. Previous studies have found that trait dynamics in response to stress or warming are governed by eco-evolutionary processes, which often determine the rates and magnitude of trait evolution [83,84] in response to warming. Different traits may evolve slower or faster with increasing temperature, which in turn can advance tipping or delay recovery [36,85]. Future research on eco-evolutionary interactions within the temperature-dependent mutualistic network model can increase our understanding of warming-induced tipping.

## Data Availability

Codes and data are available in a Zenodo repository (https://doi.org/10.5281/zenodo.7220522) [86]. The data are provided in electronic supplementary material [87].
